# Augmented Reality in Extratemporal Lobe Epilepsy Surgery

**DOI:** 10.3390/jcm13195692

**Published:** 2024-09-25

**Authors:** Alexander Grote, Franziska Neumann, Katja Menzler, Barbara Carl, Christopher Nimsky, Miriam H. A. Bopp

**Affiliations:** 1 Department of Neurosurgery, University of Marburg, Baldingerstrasse, 35043 Marburg, Germany; franziska.neumann@uk-gm.de (F.N.); barbara.carl@helios-gesundheit.de (B.C.); nimsky@med.uni-marburg.de (C.N.); 2 Department of Neurology, University of Marburg, Baldingerstrasse, 35043 Marburg, Germany; katja.menzler@med.uni-marburg.de; 3 Department of Neurosurgery, Helios Dr. Horst Schmidt Kliniken, Ludwig-Erhard-Straße 100, 65199 Wiesbaden, Germany; 4 Center for Mind, Brain and Behavior (CMBB), 35043 Marburg, Germany

**Keywords:** epilepsy surgery, focal cortical dysplasia, extratemporal lobe epilepsy, multimodality, neuronavigation, augmented reality, AR

## Abstract

**Background**: Epilepsy surgery for extratemporal lobe epilepsy (ETLE) is challenging, particularly when MRI findings are non-lesional and seizure patterns are complex. Invasive diagnostic techniques are crucial for accurately identifying the epileptogenic zone and its relationship with surrounding functional tissue. Microscope-based augmented reality (AR) support, combined with navigation, may enhance intraoperative orientation, particularly in cases involving subtle or indistinct lesions, thereby improving patient outcomes and safety (e.g., seizure freedom and preservation of neuronal integrity). Therefore, this study was conducted to prove the clinical advantages of microscope-based AR support in ETLE surgery. **Methods**: We retrospectively analyzed data from ten patients with pharmacoresistant ETLE who underwent invasive diagnostics with depth and/or subdural grid electrodes, followed by resective surgery. AR support was provided via the head-up displays of the operative microscope, with navigation based on automatic intraoperative computed tomography (iCT)-based registration. The surgical plan included the suspected epileptogenic lesion, electrode positions, and relevant surrounding functional structures, all of which were visualized intraoperatively. **Results**: Six patients reported complete seizure freedom following surgery (ILAE 1), one patient was seizure-free at the 2-year follow-up, and one patient experienced only auras (ILAE 2). Two patients developed transient neurological deficits that resolved shortly after surgery. **Conclusions**: Microscope-based AR support enhanced intraoperative orientation in all cases, contributing to improved patient outcomes and safety. It was highly valued by experienced surgeons and as a training tool for less experienced practitioners.

## 1. Introduction

In the neurosurgical application, augmented reality (AR) superimposes virtual information into the surgeon’s view of the patient and thereby complements and integrates the concept of standard surgical navigation relying on only virtual reality, providing a 3D virtual model including anatomical and functional relevant information overlaid on the surgical field [1]. First proposed by Kelly et al. [2] and Roberts et al. [3] in the 1980s, the injection of overlays of additional information provided by the imaging data into the operating microscope’s optical image served as the foundation for the further development of neurosurgical AR hardware. With the commercialization of head-up display (HUD) operating microscopes in the 1990s, microscope-based AR was introduced to a broader neurosurgical community [4,5]. Microscope-based AR support has often been applied in tumor surgery, allowing for real-time AR visualization of target and structural and functional risk structures [6,7,8,9,10,11]. With further availability of state-of-the-art operating microscopes integrated into the navigation systems, this technique was also applied to skull base, vascular, and spine surgery [12,13,14,15].

Epilepsy affects almost 65 million people worldwide, of whom one-third are termed pharmacoresistant [16,17,18]. Whereas temporal lobe epilepsy (TLE) is the most common type of pharmacoresistant epilepsies [19], in 20% of patients, epileptogenic activity is localized extratemporal [20]. Surgery is an established treatment for carefully selected patients who have pharmacoresistant epilepsy, especially in TLE [19], with excellent patient outcomes [21]. However, extratemporal lobe epilepsy (ETLE) poses significant challenges to epileptologists and neurosurgeons [22,23,24]. In those cases, non-invasive diagnostics are often limited, especially when radiological assessments, seizure semiology, and neuropsychology are equivocal [25]. Thus, further invasive diagnostics (depth electrodes/subdural grid electrodes) are required to precisely identify and localize the epileptogenic focus, with boundaries not necessarily defined anatomically, and the involvement of eloquent cortical areas and to develop an optimal surgical plan to increase postoperative seizure freedom [22,24,25,26,27,28].

Neuronavigation support has also proven to be a beneficial tool in epilepsy surgery, allowing for a correlation of imaging data, additional information gained from multimodal diagnostics, and patient data space [24,29,30,31,32]. In epilepsy surgery of clearly identifiable lesions, navigation might serve to tailor craniotomy and maximize the extent of resection while minimizing the risk of functional impairment, as compared to cranial tumor surgery. However, in the case of indistinct lesions that might also be not easily identifiable macroscopically, subtle cortical dysplasia, or epileptogenic focus not related to any anatomical lesion, image guidance is of particular importance to improve patient outcome in terms of seizure freedom concerning eloquent cortical areas. It can also be used during invasive diagnostics and resection for optimized intracranial electrocorticography (EEG) electrode positioning [29].

However, common standard navigation is used via separate navigation displays close to the surgical field, requiring dedicated navigation instruments (e.g., pointer). This raises the need to switch surgical instruments and alternate viewing directions from display to patient and vice versa throughout the surgery [33]. Virtualizing the physical instruments’ tooltip using the microscope’s focal point and integrating all relevant information into the surgical view, microscope-based AR enhances the surgeon’s mental visualization of the navigation data, easing orientation, lowering the demand for attention shifts, and increasing surgeon comfort [1,33,34]. This might be particularly interesting in ETLE, including information from invasive diagnostics to identify the epileptogenic focus and eloquent tissue without clear anatomical boundaries.

Therefore, the present study aimed to report on the usability, practicability, and clinical experience of microscope-based AR support in ETLE surgery. Up to now, there are only rare reports on the use of neuronavigation and AR assistance in non-lesional ELTE surgery. Here, we report on a carefully selected cohort of ETLE patients who underwent invasive diagnostics followed by navigation and AR-supported resection of the supposed lesion. 

## 2. Materials and Methods

### 2.1. Study Cohort

Within this study, data of ten patients with ETLE who underwent surgery between September 2016 and June 2024 were analyzed. Patients were included who (1) presented with ETLE, (2) underwent invasive diagnostics using stereo EEG (SEEG) depth electrodes and/or grid electrodes, and (3) underwent consecutively neuronavigation- and microscope-based AR-supported resection of the suspected epileptogenic lesion. All patients who underwent only invasive diagnostics or surgery without invasive diagnostics due to lesions that could be clearly identified and outlined within the imaging data (e.g., tumors) were excluded. Ethics approval for prospectively collecting routine clinical and technical data during neurosurgical treatment of patients was obtained in accordance with the Declaration of Helsinki by the local ethics committee at the University of Marburg (No. 99/18); a retrospective analysis of the collected data was also permitted by the ethics committee (RS 24/214). Written informed consent was provided by all included patients.

All patients initially underwent presurgical assessments following a standard protocol incorporating clinical, imaging, neuropsychological, and EEG data. During the interdisciplinary discussion (epileptology, neurosurgery, neuropsychology, neuroradiology), surgery for invasive diagnostics using SEEG and/or subdural grid electrodes was indicated, mainly including the patient with a not clearly localizable epileptogenic lesion; mapping of the surrounding eloquent cortex was requested due to resectability of the lesions or verification of an identified lesion and seizure onset. The same procedure was implemented after invasive interdisciplinary diagnostics, indicating the respective surgery for the suspected epileptogenic lesion.

### 2.2. Preoperative Imaging and Planning for Invasive Diagnostics

After initial diagnostic imaging, all patients underwent preoperative MRI imaging using a 3T MRI system (Tim Trio, Siemens, Erlangen, Germany) equipped with a 12-channel head matric Rx-coil. Data acquisition included a 3D T1-weighted, 3D T2-weighted, 3D fluid attenuated inversion recovery (FLAIR), time-of-flight (ToF) angiography data set as well as a diffusion-weighted (DWI) single-shot echo planar imaging (EPI) data set for fiber tractography with 30 non-co-linear diffusion encoding gradients (high b-value 1000 s/mm^2^). If applicable, functional MRI (fMRI) data for localization of Broca’s and Wernicke’s area were assessed using silent word generation, semantic decision, and passive listing tasks. 

After rigid image co-registration of all required and available data sets using the image fusion element (Brainlab, Munich, Germany), SEEG trajectories were manually planned using the trajectory element (Brainlab, Munich, Germany). MRI-positive lesions were manually outlined using the smart-brush element (Brainlab, Munich, Germany), as well as vascular risk structures. In addition, and depending on the localization of the suspected lesion, various anatomical structures such as the amygdala or the hippocampus were automatically segmented using the anatomical mapping or object manipulation element (Brainlab, Munich, Germany), if necessary being manually refined to match the individual patient anatomy. 

Depending on the localization of the lesion, fiber tractography of different major white matter tracts, such as the corticospinal tract, arcuate fascicle, and optic radiation, were reconstructed using the fiber tracking element (Brainlab, Munich, Germany) with a standard diffusion tensor imaging-based deterministic approach. If applicable, fMRI data were analyzed using SPM8/SPM 12 following a standard protocol (without normalization), and resulting activation clusters were incorporated into the preoperative plan.

### 2.3. Operating Room Setup

All patients underwent frame-based (SEEG) or frameless (SEEG, grid, resection) navigation-supported surgery for invasive diagnostics or resection. Therefore, the operating room was equipped with a neuronavigation system (Curve Navigation, Brainlab, Munich, Germany), a mobile 32-slice intraoperative CT (iCT) system (AIRO^®^, Brainlab, Munich, Germany), operating microscopes (Pentero 900 or Kinevo 900, Zeiss, Oberkochen, Germany), ultrasound systems (FlexFocus 800/BK5000, BK Medical, Herlev, Denmark) fully integrated into the navigation system, and a stereotactic frame (Zamorano-Dujovny (Inomed, Emmendingen, Germany) and the frameless stereotactic VarioGuide system (Brainlab, Munich, Germany).

### 2.4. Intraoperative Workflow for Invasive Diagnostics

SEEG electrodes were initially implanted in a frame-based stereotactic procedure as described by [35]. Alternatively, in the later cases, SEEG electrodes were implanted in a frameless stereotactic procedure using the VarioGuide system. For this purpose, an initial registration iCT scan was performed in the OR for frame localization and final calculation of trajectory coordinates or patient registration and integration of the corresponding VarioGuide parameters. After sequential implantation of depth electrodes (ADtech, did medical, Simbach am Inn, Germany), a second iCT scan was performed to verify the electrodes’ spatial location and to rule out early surgical complications.

**Figure 1:**
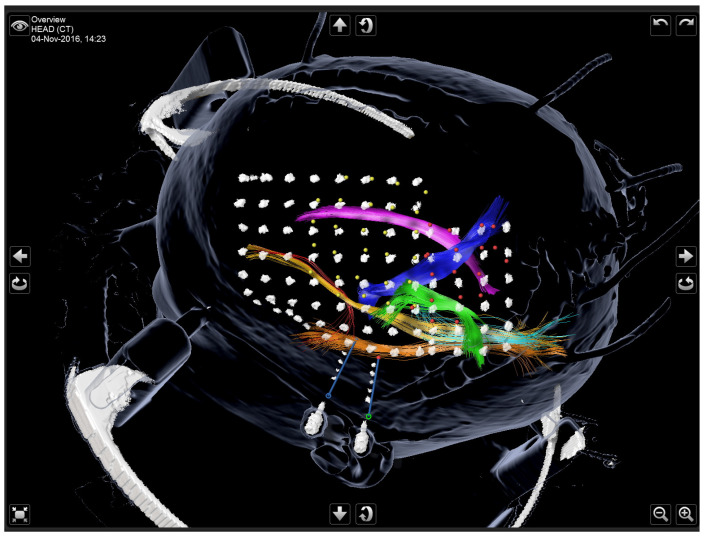
3D Visualization after the second iCT scan showing two SEEG electrodes in relation to their intended location (blue lines), two subdural grid electrodes reconstructed from iCT data, and intraoperatively acquired points corresponding to the intraoperatively accessible electrode contacts (yellow and red dots) using the navigated operating microscope, as well as major left hemispheric white matter tracts.

Subdural grid electrodes were implanted in a frameless navigation-supported manner. Therefore, an initial iCT-based registration scan was performed following our institutional procedure [36]. After standardized skin incision and navigation-controlled craniotomy, the subdural grid electrodes (ADtech, did medical, Simbach am Inn, Germany) were placed on the desired area of interest of the exposed cortex. The wires were tunneled under the skin and fixated by suture. Afterward, all accessible electrode contacts were acquired as points using the navigated operating microscope and labeled accordingly to map EEG data. After surgical closure, a second iCT scan was performed to identify the subdural grid electrode pattern and to rule out early surgical complications (see Figure 1).

### 2.5. Preoperative Imaging and Planning for Resection

Following invasive monitoring and after analyses of all EEG data, the results were discussed interdisciplinarily again. The planned extent of resection and localization of functional risk structures according to stimulation results were determined interdisciplinarily. Depending on the time interval between SEEG/grid explantation and resection, the already existing surgical plans, including the intraoperative postsurgical CT scan, were used or updated with new structural MRI data acquired inbetween in the clinical routine.

**Figure 2:**
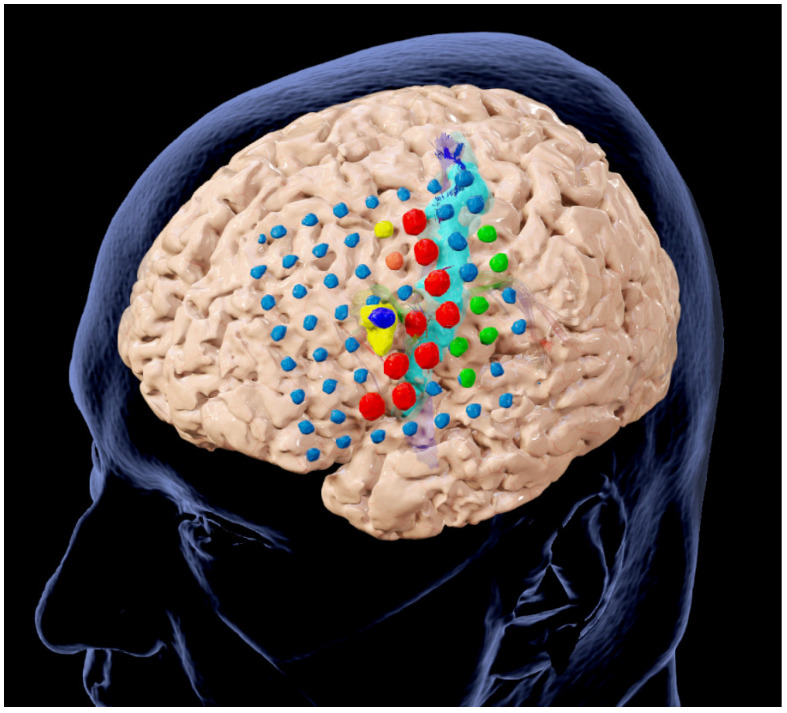
Preoperative planning after invasive diagnostics, including segmentations of the cerebrum, the motor cortex (light blue), grid electrodes (blue) and SEEG electrode (dark blue), functional areas according to stimulation (green, red, orange), the epileptogenic lesion (yellow), and white matter tracts (corticospinal tract, arcuate fascicle) in close vicinity to the target lesion.

Following the initial multimodal surgical plans, including outlined structures and risk structures and, if applicable, major white matter tracts and language-related activation clusters from fMRI data, all electrode contacts (SEEG and grid) were segmented using a threshold-based approach (Smart Brush, Brainlab, Munich, Germany). All electrode contacts dedicated to the seizure onsets were additionally outlined for intraoperative visualization, and, if possible, a 3D object covering the intended extent of the resection was manually generated (see Figure 2).

### 2.6. Intraoperative Workflow for Resection of the Epileptogenic Lesion

Under general anesthesia, if present, SEEG electrodes were removed, and the patient’s head was fixated in a radiolucent carbon head clamp (DORO, Black Forest Medical Group, Freiburg, Germany) using three metallic pins. Whereas pin-related artifacts are less of an issue for the low-dose registration scan, for potential full-dose control scans at the end of resection, the pins were placed in a way that the relevant area can be visualized without metallic artifacts (typically beyond and above the area of interest). For navigational purposes, a radiolucent patient reference geometry was mounted at the head clamp’s left side, and three adhesive skin markers were attached to the patient’s head for assessment of registration accuracy.

For automatic intraoperative patient registration, a sequential low-dose iCT scan was performed (7.1 mA, 120 kV, 1.92 s exposure time, 1 mm reconstructed slice thickness, 512 × 512 matrix size, 33.3 cm^2^ field of view) covering 6.2 cm, resulting in a dose-length product of 17.8 mGy*cm). The target registration error (TRE) was calculated as offset between the physical pointer’s tooltip that was placed in the divot of the three skin markers and the virtualized tooltip in the digital representation of the markers in the acquired iCT data set. After high patient registration accuracy was verified, the preoperative planning data were rigidly co-registered with the low-dose registration iCT scan, allowing immediate navigation support.

In cases where only SEEG electrodes were implanted for invasive diagnostic and, therefore, were removed before the recent surgical intervention, standardized skin incision and navigation-controlled craniotomy were performed to assess the epileptogenic lesion. Sutures and the bone flap were removed if subdural grid electrodes were used. In the latter case, the dura was opened again, and before the removal of the subdural grid electrodes, navigation accuracy was assessed using outlines of the segmented electrode contacts visualized within the microscope; if necessary, a microscope-based navigation update was performed. Afterward, the subdural grid electrode was removed. At this stage, or in cases where no subdural grid electrodes were used, before durotomy, navigated intraoperative ultrasound (iUS) using a craniotomy transducer (imaging depth: 65 mm) was performed to assess navigation accuracy by overlaying MRI-based object outlines onto the live-ultrasound view and to further identify the lesion within the ultrasound data, if possible, for intraoperative resection control. For this purpose, besides navigated live-ultrasound usage, a 3D iUS data set was acquired by constantly sweeping the probe across the accessible dural (SEEG) or cortical (grid) layer. In the case of cortical lesions, a linear high-frequency ultrasound probe was used in addition to gain high-resolution images of the suspected epileptogenic lesion. Navigation-supported resection wasthen performed using microscope-based AR support. 

### 2.7. Augmented Reality

Microscope-based AR support was utilized with the head-up displays (HUD) of fully integrated operating microscopes Pentero 900/Kinevo 900 (Zeiss, Oberkochen, Germany) without any need for further AR supporting devices such as head-mounted displays or specific glasses. The microscope was tracked in the navigational space using an attached four-sphere registration array to allow for AR support throughout the surgery.

The AR visualization was calibrated before its use to overcome minor spatial initial misalignments. All outlined objects, such as relevant electrode contacts, lesions, and vascular, functional, and structural risk structures related to the surgical target, can then be visualized using the AR display. Therefore, the integrated HUD superimposes the 3D objects in the operating microscope. In parallel to microscope-based AR support, fused multimodal image data enriched with outlined objects and structures are visualized on the navigation displays (Navigation, Brainlab, Munich, Germany) close to the surgical field. 

### 2.8. Surgical and Epileptogenic Outcomes

Each patient’s surgical and neurological (transient and persistent) outcomes were assessed after surgery. Postoperative outcomes concerning epileptic seizures after surgery were assessed using the International League Against Epilepsy (ILAE) outcome classification [37] at in-house follow-up examinations or during structured telephone interviews.

## 3. Results

### 3.1. Clinical and Demographic Information

This study included ten patients (mean age: 33.80 ± 12.21 years, male/female: 7/3). Four patients presented with left frontal lobe epilepsy, one with left/bilateral frontal lobe epilepsy, two with right frontal lobe epilepsy, one with right frontotemporal lobe epilepsy, one with parietal lobe epilepsy (recurrence epilepsy surgery), and one with left hemispheric epilepsy. All patients underwent invasive video-EEG monitoring in preparation for resective epilepsy surgery; in three cases, solely SEEG electrodes, and in three cases, solely subdural grid electrodes were implanted. In the remaining four cases, SEEG and subdural grid electrodes were used (two cases: parallel, two cases: sequential) for invasive diagnostics. After invasive video-EEG monitoring, all patients underwent tailored resection accordingly. Neuropathological examination of the resected tissue revealed focal cortical dysplasia (n = 5), diffuse neuronal heterotopia (n = 3), gliotic scars (n = 1), and hypoxic tissue (n =1), see Table 1 and Table 2). 

### 3.2. Surgical and Epileptogenic Outcomes

Of the then patients, one patient showed a dislocation of the bone flap that was refixed; another one showed a cerebrospinal fluid (CSF) fistula that needed revision surgery, including a dural patch and subsequent CSF drainage. One patient showed a slight coordination disturbance of the left side, which declined within one month after surgery, and another patient presented with a mild transient sensory aphasia postoperatively, which entirely recovered within three weeks after surgery. 

At the time of the 1-year follow-up, six patients (60%) presented with no further seizures (ILAE class 1), one patient was attributed to ILAE class 2, two patients were assigned to ILAE class 3, and one was assigned to ILAE class 4. During long-term follow-up with varying time intervals, all patients initially assigned to ILAE class 1 remained seizure-free; one patient, initially ILAE class 3, reported no further seizures at the last follow-up (ILAE class 1, 2 years postoperative); the remaining three patients had unchanged seizure outcome compared to the one-year follow-up. At the time of the last follow-up, therefore, 70% of the patients were seizure-free. For further details, see Table 3.

### 3.3. Navigation and Augmented Reality Support

Navigation and microscope-based AR support was facilitated in all surgeries with a mean TRE of 0.75 ± 0.26 mm. Depending on the individual cases, the suspected epileptogenic lesion identified or confirmed by invasive video-EEG monitoring, information about SEEG electrode and/or subdural grid electrode positions, relevant electrode contacts related to the recorded seizure onsets, relevant surrounding functional structures (e.g., motor cortex), major white matter tracts, and, in some cases, cortex representations were included in the surgical plan and visualized throughout the surgery. Details on the included structures can be found in Table 4. All structures could thereby be individually switched on and off to provide efficient and tailored AR support throughout the surgery. Microscope-based AR support improved intraoperative orientation in all cases and contributed to patient safety while increasing the surgeon’s comfort.

### 3.4. Illustrative Case (Patient No. 4)

Patient No. 4 (male, 30 years old at the time of surgery), first diagnosed in the second year of his life, presented with right parietal lobe epilepsy. Seizures, most often out of sleep, showed sensitive auras of the left hand, followed by tonic and clonic convulsions of the left arm and face. MR imaging revealed no pathological alterations. Four years earlier, the patient had already undergone invasive video-EEG monitoring using a subdural grid electrode placed across the postcentral gyrus, followed by resection of the suspected epileptogenic lesion in the parietal lobe. Neuropathological examination revealed a potential FCD type I or type IIb (no precise classification possible). After one year of seizure freedom, the patient presented again with the same seizure semiology despite anticonvulsive medication (current medication: carbamazepine, brivaracetam, lacosamide; previous discontinued ineffective medication: levetiracetam, zonisamide). Repeated MRI showed the resection cavity surrounded by slight gliosis but no further pathological alterations. FDG-PET-CT likewise revealed no pathological findings. Interdisciplinary discussion led to the indication for further invasive video-EEG monitoring with SEEG electrodes around the old resection cavity.

Five SEEG electrodes were implanted in a frameless navigation setup as described above. During invasive video-EEG monitoring, 22 seizures were recorded, with seizure onset at the anterior SEEG electrode within the postcentral gyrus and late mesial seizure propagation stimulation along the anterior SEEG electrode. The seizures showed slight paresthesia of the left arm, body, and leg. After an interdisciplinary discussion, the epilepsy surgery board recommended surgical removal of the identified region at the anterior border of the resection cavity.

**Figure 3:**
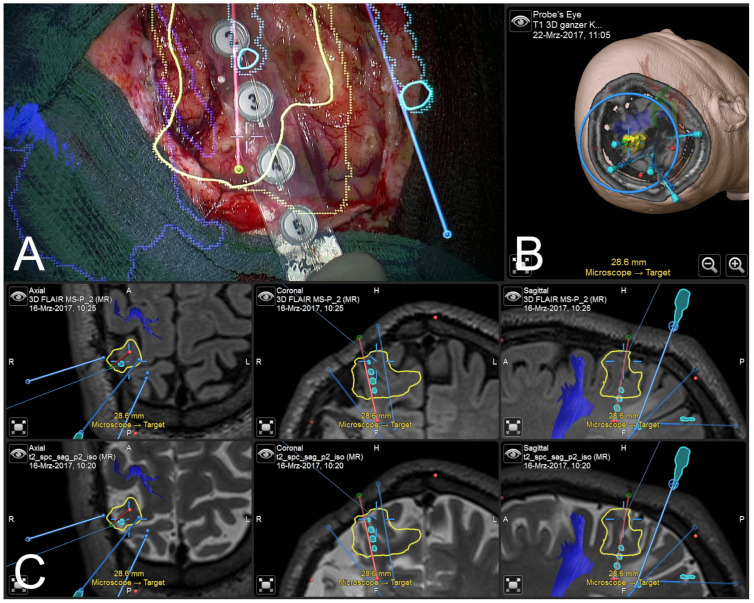
Microscope-based AR visualization of outlined epileptogenic focus (yellow), segmented SEEG contacts (light blue), planned SEEG trajectories (blue and red), reconstructed corticospinal tract (blue) during ECoG before resection (**A**), in probe’s eye 3D view (**B**), and in axial, coronal, and sagittal (left to right) navigation views using FLAIR and T2 weighted MRI data sets (**C**).

Surgery was performed under general anesthesia. Following automatic patient registration using the intraoperative CT, rigid fusion of preoperative planning data, including outlines of the SEEG electrodes, outlines of the intended extent of resection in the postcentral gyrus, and surrounding functional risk structures such as the corticospinal tract, was conducted. According to the localization of the epileptogenic lesion, the previous craniotomy was extended. Before durotomy, applied intraoperative navigated ultrasound showed high navigation accuracy and visualized the lesion. After durotomy, electrocorticography was performed using a six-contact strip electrode, immediately showing high epileptogenic activity across the outlined lesion (see Figure 3).

Following the outlines of the epileptogenic focus along the initial SEEG electrode, the epileptogenic tissue is resected. Intermittent electrocorticography (ECoG) along the extended resection cavity still showed epileptogenic activity. Therefore, the resection was extended laterally. Finally, ECoG revealed no further lateral, mesial, or dorsal epileptogenic activity or such activity within the resection cavity.

Neuropathological examination diagnosed focal cortical dysplasia type IIa. Postoperatively, the patient showed no new focal neurological deficits. A slight coordination disturbance of the left side was identified during early postoperative physiotherapeutic treatment, declining within one month.

One year after surgery, the patient reported no further seizures (ILAE 1). Without anticonvulsive therapy, the patient experienced one seizure during the second year after surgery. An anticonvulsive therapy (valproate) was prescribed again. Since then, the patient reported no further seizures (5-year follow-up: ILAE 1, latest follow-up (7 years) ILAE 1).

### 3.5. Illustrative Case (Patient No. 9)

Patient No. 9 is a 23-year-old male patient, first diagnosed at seven. The patient presents with left frontal lobe epilepsy with sleep related seizures, including tonic contractions of the arms, dialeptic and astatic seizures, and seizures with hyperventilation. Initial presurgical video-EEG monitoring at the age of 16 was repeated at the age of 22 due to a consistently high seizure frequency despite anticonvulsive medication, including sulthiame (discontinued due to an increase in seizure frequency), oxcarbazepine (discontinued), and recently brivaracetam, lacosamide, and perampanel. Presurgical video-EEG monitoring recorded 27 seizures with seizure onset, if localizable, at F3. MRI imaging revealed a suspected cortex thickening in the left superior frontal gyrus (parafalxial and superior of the left cingulate gyrus), whereas FDG-PET-CT showed no pathological findings. Based on these inconclusive findings, interdisciplinary discussion of the case indicated bifrontal SEEG implantation for invasive EEG diagnostics.

Eight SEEG electrodes were implanted using the Varioguide system for precise placement, confirmed by intraoperative CT imaging. The monitoring recorded 57 seizures from a specific SEEG electrode, correlating with the radiologically diagnosed cortical thickening in the superior frontal gyrus. Due to the proximity to Broca’s area and the motor cortex, further diagnostic procedures were needed to determine the extent of cortical resection. Therefore, an 8 × 8 subdural grid electrode was implanted, covering Broca’s area and the nearby motor cortex. Invasive monitoring identified 19 additional focal seizures near the previously identified SEEG electrode’s location. Cortical stimulation identified motor and language areas, revealing critical functions at some seizure onset points. Given the risk of functional impairment, the epilepsy surgery board recommended a targeted resection.

According to the information gained, a presurgical plan was generated, including multimodal preoperative MRI data and intraoperative CT data after SEEG and subdural grid electrode implantation. Within the MRI data, the suspected cortical thickening, the to-be-resected tissue surrounding the relevant SEEG electrode contacts (one to three), and the motor cortex were outlined manually, and relevant major white matter tracts close by—the corticospinal tract and the arcuate fascicle—were reconstructed. In addition, the cerebrum was segmented automatically for navigation update purposes. Within the iCT data sets, on the one hand, the subdural grid electrodes’ contacts, as well as the SEEG electrode’s contacts, were segmented using a threshold base approach, followed by individual segmentation of the recorded seizure onset contacts as well as contact positions related to the identified motor cortex, sensory cortex, and areas related to language production.

**Figure 4:**
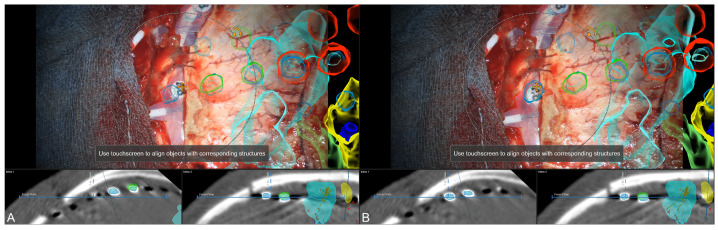
Microscope-based navigation update using segmented outlines of the subdural grid electrodes (blue) increased overall navigation accuracy ((**A**): slight mismatch at contact level, (**B**): compensation of mismatch by translation of image data). Visualization of various objects and structures in the recent microscope’s focal plane, such as motor cortex (light blue), epileptogenic tissue (yellow and light green), epileptogenic focus according to SEEG (dark blue), subdural grid electrodes (red), and sensory areas according to stimulation (green).

Surgery was performed under general anesthesia. Automatic patient registration was performed using the intraoperative CT, showing a high initial registration accuracy, followed by a rigid co-registration of iCT and preoperative planning data. After craniotomy and before opening the dura again, a microscope-based navigation update was performed using the outline contacts (see Figure 4). 

**Figure 5:**
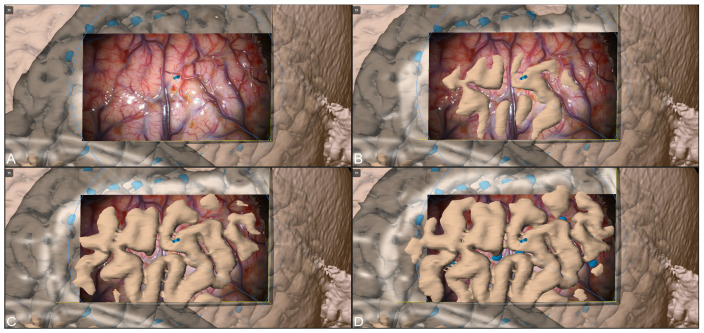
AR-based verification of high navigation accuracy using a 3D visualization of the segmented cortex after microscope-based navigation update while sequentially (**A**–**D**) shifting the microscope’s focal plane along the focal axis.

After durotomy and removal of the subdural grid electrode, navigation accuracy was again verified using a 3D cortex representation of the operating microscope by sequentially shifting the microscope’s focal plane along the focal axis, showing a good match between 3D visualization and the surgical view (see Figure 5).

**Figure 6:**
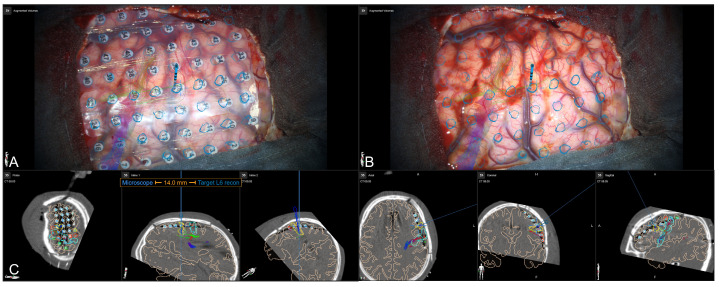
Microscope-based AR visualization of outlined subdural grid electrode contacts, lesion-associated SEEG trajectory and the corticospinal tract before (**A**) and after grid removal (**B**) with in-parallel view of the navigation display (**C**) in probe’s eye view, inline views, and standard axial, coronal, and sagittal views.

**Figure 7:**
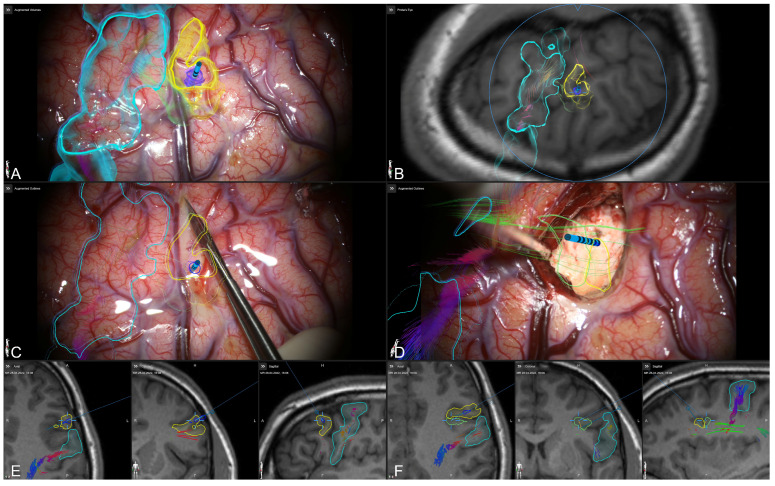
Microscope-based AR support throughout the surgery. Three-dimensional visualization of outlined structures (motor cortex: light blue, epileptogenic tissue: yellow and light green, epileptogenic focus according to SEEG: dark blue), SEEG trajectory, and major white matter tracts (corticospinal tract, arcuate fascicle) after durotomy and grid removal within the microscope view (**A**) and in probe’s eye view (**B**). Two-dimensional visualization of all structures using microscope-based AR support (**C**,**D**) and in-parallel standard navigation views (**E**,**F**), at the beginning of corticotomy (**C**,**E**) and at the end of resection (**D**,**F**).

Despite standard navigation displays, allowing for a mental transfer of image data and outlined structures onto the situs, the usage of microscope-based AR allowed for an eased and intuitive intraoperative guidance, as the subdural grid electrodes’ positions, as well as all grid-related outlines, were still available in the surgeon’s viewing trajectory after grid and SEEG electrode removal (see Figure 6) before resection, as well as during and after resection (see Figure 7). 

Neuropathological examination of the resected tissue revealed a diffuse neuronal heterotopia. Postoperatively, the patient presented with transient sensoric aphasia that recovered entirely within three weeks. In addition to two seizures immediately after surgery, no further seizures were recorded at the one-year follow-up (ILAE 3). No further seizures were reported at the latest follow-up (2 years, ILAE 1). 

## 4. Discussion

The scope of this study was to prove the clinical and educational benefits of neuronavigation support and in particular microscope-based AR assistance in non-lesional ETLE surgery. Microscope-based AR support was facilitated in all cases and improved intraoperative orientation and contributed to patient safety while increasing the surgeon’s comfort. Therefore, the results of this study support the hypothesis of clinical advantages of microscope-based AR support in the surgical treatment of ETLE patients.

Epilepsy surgery is a well-established treatment option for carefully selected patients with pharmacoresistant epilepsy. One major prerequisite for its success is the ability to clearly and precisely identify and resect the epileptogenic zone without hampering neurological function [21,31], which can pose a significant challenge to epileptologists and neurosurgeons [22,23,24]. Clearly and precisely identifying this epileptogenic zone might be particularly provoking in ETLE, which is only present in roughly 20% of patients with pharmacoresistant epilepsy [20]. With its tremendous evolution, MRI has become the most essential imaging modality in the radiological assessment of epileptogenic lesions. Nevertheless, even with increasing high-field and high-resolution imaging and advanced post-processing algorithms, still, in roughly one-third of the patients with pharmacoresistant epilepsy, no structural alternation is radiologically seen, which is especially often the case in ETLE patients in comparison to TLE patients [38,39].

Besides non-lesional MRI in many cases, ETLE is often characterized by nonspecific seizures, a fast spread of epileptogenic potentials, and larger epileptogenic zones also involving eloquent cortical regions. In this way, often not only in MRI-negative cases but also in MRI-positive cases, no unequivocal hypothesis for resectability and extent of resection can be generated non-invasively [25]. Complete resection of the epileptogenic zone while preserving functional integrity is one significant predictor for postoperative seizure freedom [40,41,42,43]. Therefore, an invasive workup is required to discuss the overall option for surgery and evolve a suitable surgical plan to achieve and predict chances for postoperative seizure freedom [22,24,25,26,27,28,29].

Especially in those cases of pharmacoresistant epilepsy, invasive EEG is a well-established part of presurgical diagnostics [28,44]. In this way, invasive diagnostics with depth and/or subdural grid electrodes can be utilized, on the one hand, to identify and localize the epileptogenic focus, with its boundaries not necessarily defined anatomically or easily seen on a macroscopic level and, on the other hand, to evaluate the involvement of close-by eloquent cortical areas [24,29]. Invasive EEG, thereby, has been shown to be suitable for identifying and delineating seizure onset zones in MRI-positive and MRI-negative cases [45]. However, in the case of indistinct lesions that might also be not identifiable macroscopically, subtle cortical dysplasia, or epileptogenic focus not related to any anatomical lesion, image guidance is of particular importance to improve patient outcome in terms of seizure freedom with respect to eloquent cortical areas. It can also be used during invasive diagnostics and resection for optimized positioning of intracranial EEG electrodes [29]. 

Since its development and introduction in the 1990s, neuronavigation and microscope-based AR have become inevitable supportive tools for various neurosurgical procedures, including tumor, vascular, and spinal surgery [46,47,48]. Precise planning for the surgical approach and identifying the spatial relationship of the target and surrounding risk structures as prerequisites for its application contribute to surgical preoperative and intraoperative decision-making and support intraoperative surgical orientation and further radical resection while increasing patient safety [49,50,51,52,53,54]. In this way, neuronavigation has also found its way into functional neurosurgery and epilepsy surgery in terms of planning, invasive diagnostics, and resection of epileptogenic zones [24,29,30,31,32]. Neuronavigation support thereby allows for an accurate resection of the epileptogenic zone, especially in cases with lesions that cannot be distinguished macroscopically from adjacent healthy surrounding brain tissue, such as, e.g., cortical dysplasia [24,55,56].

So far, only a few studies have investigated the role of navigation support in ETLE. As only a minor percentage of pharmacoresistant epilepsy patients present with ETLE, most often small patient cohorts are reported, some of them also including TLE patients [31,45,57]. In an early work by Wurm et al. [24], including a broad variety of epilepsy patients (MRI-positive (e.g., tumor, malformations) and MRI-negative), it was shown that navigation support led to individual tailoring of craniotomy and corticotomy, precise targeting of small or deep-seated surgical targets, accurate placement of electrodes for invasive diagnostics, advanced multimodal planning also integrating clinical and EEG data, and further safe manipulation close to sensitive areas. Sommer et al. [57,58] reported on the use of magnetoencephalography, functional neuronavigation, and intraoperative MRI in ETLE and further specifically in FLE patients and showed extended resection rates while preserving functional integrity with favorable seizure outcomes and acceptable moderate neurological impairment in specifically complex cases of ETLE. However, they also elaborated on the specific value of iMRI in terms of resection control and in terms of loss of navigation accuracy due to brain shift in the course of surgery. In a technical report by Chamoun et al. [29], navigation-guided placement of subdural electrodes is suggested. Especially in the case of non-anatomically identifiable lesions, as often seen in ETLE, it is crucial to delineate the epileptogenic lesion and the functional surrounding tissue clearly, and it can also be used to guide resection. As stated there and in another technical report by Kamida et al. [30], implanted electrodes, identifiable on postoperative imaging data, could also be visualized to keep this spatial information after removal of those during resection and not impede resection. A recent study reporting on multimodal planning and intraoperative integration using navigation and intraoperative MRI in a small cohort of MRI-negative ETLE patients concluded that those patients can be treated successfully when extensive preoperative planning is performed.

A previous report on a large patient cohort stated that seizure freedom postoperatively was seen in only 35% of patients with MRI-negative epilepsy and 60% in the case of MRI-positive patients; however, these included TLE and ETLE patients [59]. Maslarova et al. reported seizure freedom in MRI-negative ETLE patients in 71% of the cases [31]; another report revealed seizure freedom of 61% in MRI-negative and 64% in MRI-positive FLE patients with the use of navigation support [58]. With navigation and microscope-based AR support in the current study, overall seizure freedom postoperatively was reached in six patients (60%) at the one-year follow-up and in seven patients (70%) at the latest follow-up (median 55.50 months). Three of the four MRI-negative patients postoperatively reported no seizures (75%), comparable to [31,58] utilizing navigation and intraoperative imaging. In the remaining six MRI-positive patients, three patients at one-year follow-up (50%) and four patients (66%) at the latest follow-up reported no seizures, which is in line with previous reports [57,58]. However, due to the small sample size in this study and previous studies, those results need to be interpreted with care.

However, common standard navigation is used via separate navigation displays close to the surgical field, requiring dedicated navigation instruments (e.g., pointer), raising the need to switch surgical instruments and alternating viewing directions from display to patient and vice versa throughout the surgery [33]. Virtualizing the physical instruments’ tooltip by using the microscope’s focal point and integrating all relevant information into the surgical view, microscope-based AR enhances the surgeon’s mental visualization of the navigation data, easing orientation, lowering the demand for attention shifts and thereby also increasing surgeon comfort [1,33,34]. This might be particularly interesting in ETLE, including information from invasive diagnostics to identify the epileptogenic focus and eloquent tissue without clear anatomical boundaries. However, only one study explicitly used a microscope integration with projections of outlined data. This study investigated a patient cohort with apparent temporal and extratemporal lesions (e.g., tumor, vascular malformations, gliosis) without the need for invasive diagnostics, providing guidance in situ with high reliability for complete resection of the lesion [60].

Prior implementations of AR support using the operating microscope superimposed manually outlined objects by dashed lines in the recent focal plane of the microscope being perpendicular to the viewing axis, with the help of the microscope’s HUD [10,61,62]. However, as the dashed outlines represent the perimeter of the segmented structures two-dimensionally (2D), depth perception, crucial in various parts of the surgery, might be limited, especially when addressing deep-seated structures [63,64]. Following the tremendous evolution in this field, the recent state-of-the-art implementation of microscope-based AR support allows for an improved three-dimensional (3D) perception of outlined structures superimposed onto the surgical view. Improved HUD resolution, the use of multiple colors to discriminate objects, and a smooth real-time visualization due to massively increased computing power and efficient implementations contribute to further intuitive use of microscope-based AR support in various neurosurgical applications offering enhanced 2D and 3D visualization options in combination with the standard navigation displays close by. To avoid crowding of the surgical view due to increasingly complex and multiple visualizations and to adapt to the specific surgeon’s needs and demands and the recent surgical phase, the complexity of the visualization and the available visualizable objects can be separately switched on and off at any time. However, standard navigation displays can be used in parallel to provide the surgeon with context information beyond the currently visualized sectional planes [1,33].

High navigational accuracy is a prerequisite for relying on navigation and microscope-based AR support throughout surgery, especially near eloquent areas. The clinical or overall accuracy, as most relevant to the surgeon, is a mixture of application and intraoperative accuracy. Besides imaging and technical accuracy, registration accuracy mainly contributes to application accuracy [47,50]. Standard registration approaches, such as the fiducial-based registration commonly used, are strongly user-dependent, ranging from the attachment of artificial markers before image acquisition for registration purposes to the intraoperative acquisition of those landmarks using the pointer [65,66,67]. To overcome this variably low initial patient registration accuracy with reported TRE of 1.8 to 5.0 mm [48,68], intraoperative automatic registration procedures, such as iCT-based registration, allow for higher registration accuracy, as also shown in this study with mean TRE of 0.75 ± 0.26 mm.

Without high overall accuracy, navigation- and microscope-based AR support might give a false impression of security. Besides application accuracy, intraoperative accuracy plays a significant role. Accuracy is known to decline over time, e.g., due to positional shift of the patient’s head with regard to the reference, but mainly due to non-linear brain deformations during surgery caused by swelling, ongoing mass resection, loss of cerebrospinal fluid, effects of gravity, et cetera [47,48,49,51,69]. Therefore, it is important to (1) validate navigation accuracy during surgery and identify inaccuracies and to (2) compensate for those inaccuracies to gain high navigational accuracy again.

Effects of brain shift and loss of navigation accuracy can mainly be addressed by intraoperative imaging utilizing iMRI or iUS, if applicable, allowing for a (repetitively) update of navigation and partially allowing for a non-linear transformation of preoperative information onto the intraoperative data [51,54,70]. However, the use of iMRI is commonly limited due to its availability, time consumption, and high costs and is often rather used as intraoperative resection control [54,57,58,71]. In contrast, iUS and especially navigated iUS can be used repetitively and at any time during surgery as a cost-effective, quick, and straightforward-to-use tool [70,72,73]. However, microscope-based AR can also be utilized to compensate for navigation inaccuracies, as suggested by [7]. Using maximum intensity projections (MIP) of imaging data or outlines of segmented structures, navigation accuracy can be evaluated on the recent microscope’s focus plane, allowing for a translation and/or rotation of image data and objects, respectively, to match patient and image data properly. Bony landmarks (positional shift) or characteristic intracranial structures such as cortical vascular structures can be used [74]. Alternatively, 3D reconstructions overlaid onto the video frame can be utilized to assess navigation accuracy while moving the focus plane along the microscope’s optical axis. Thereby, microscope-based AR might serve as a complementary, valuable, fast, and intuitive tool to estimate the brain shift and to partially overcome these discrepancies [75]. Even though not capable of fully compensating for navigation inaccuracies, microscope-based AR support provides an additional general advantage compared to solely pointer-based navigation as the size and spatial relation of structures remain valid even though they might be spatially shifted, still allowing for intraoperative orientation.

Navigation- and microscope-based AR support might require supplementary time preoperatively due to imaging and planning and intraoperatively because of registration and calibration procedures. However, studies evaluating the extent and relevance of time needed for this setup are rare and varying in definitions of “used time” [76]. In our study group’s experience with the application of navigation and microscope-based AR support, surgical time is not significantly increased, despite a slightly increased time for patient registration, rather than for calibration of the microscope, as an add-on to a procedure without navigation support [77,78,79]. However, also in line with this [76], a significant increase in time might depend more on the familiarity of the whole OR team with the used technology. During preoperative workup, preparation of a surgical plan, including multimodal image fusion and segmentation of relevant structures, is time-consuming. Still, generating a thorough plan might also be highly recommended in the non-navigated setup for later intraoperative orientation [80]. Given the various planning tools the navigation systems offer, planning is available in a plausible amount of time, depending on image quality and user experience (clinical, technical). However, in ETLE patients, where extensive planning is required to identify and optimize the potential of respective surgery, especially in complex cases, this should not hamper its application and, anyhow, rather supports the whole process of decision-making.

Given the small group of patients who are eligible for resective surgery after extensive presurgical non-invasive and invasive workup paired with lesions often macroscopically not distinguishable from adjacent healthy surrounding brain tissue, microscope-based AR support might serve as a valuable tool for experienced surgeons, as seen in this study, enhancing surgical orientation and encouraging the mental transfer of imaging data and outlined structures onto the surgical situs, without the need for alternative viewing directions. In the same way, microscope-based AR support can also assist in educating and training residents and less-experienced surgeons. This can range from watching operative video recordings of surgeries utilizing microscope-based AR support to gaining offline experience matching image and real-world data to intraoperative applications such as assisting a surgeon, supporting mental transfer, real-time orientation, and strategies for accuracy assessment [9].

Given the results of this study, microscope-based AR support might be beneficially applied in a broad variety of surgical interventions to improve surgical outcomes, increase patient safety, and reduce morbidity. However, when using this technique, one should be aware not only of its benefits but also its limitations, e.g., its dependency on high navigational accuracy, and have strategies on hand to identify those issues and compensate for them. Therefore, it is, in our experience, recommended to use this technique not only in the complex but in particular in the “easy” cases to gain experience on its usage, applicability, and limitations. In addition, future technical developments such as non-linear image fusion of intraoperative imaging data, real-time accuracies validation checks, and updated strategies might further enhance its beneficial use in the field of neurosurgery.

## 5. Limitations

The limitations of this study include its retrospective character and the small sample size due to stringent selection criteria (only patients with ETLE who underwent invasive video-EEG monitoring followed by resection of the epileptogenic focus) to explicitly elaborate on the use of microscope-based AR support in this neurosurgical application. In addition, as also stated by other studies elaborating on the use of navigation support in this specific patient cohort, prospective controlled randomized trials would be needed. However, due to its already proven benefit in the broad neurosurgical application field with extended resections and increased patient safety, not using these techniques needs to be discussed.

## 6. Conclusions

Epilepsy surgery remains a critical intervention for patients with pharmacoresistant epilepsy, particularly when precise identification and resection of the epileptogenic zone are required. This challenge is heightened in cases of ETLE, where non-lesional MRI findings and complex seizure patterns complicate surgical planning. Invasive EEG and advanced imaging modalities, including neuronavigation and microscope-based AR, have shown promise in improving surgical outcomes. These technologies enable more accurate localization and resection of epileptogenic zones, particularly in cases where lesions are not macroscopically distinguishable.

While navigation and microscope-based AR support offer significant benefits, particularly in enhancing surgical orientation and accuracy, their application is limited by factors such as brain shift and the need for intraoperative imaging updates. 

Despite these challenges, integrating these technologies is highly valuable for experienced surgeons and training of less-experienced practitioners.

## Figures and Tables

**Table 1:** Patient characteristics.

Patient No.	Age	Sex	Epileptogenic Zone	MRI Assessment	Invasive Diagnostics	Histopathology
1	23.82	Female	FLE, left	negative	SEEG + grid	DNH
2	47.43	Male	FLE, left	FCD	SEEG	FCD type IIa
3	30.92	Male	FLE, right	FLAIR/T2 hyperintensity	SEEG + grid	FCD type IIb + ganglioglioma
4	30.82	Male	PLE, right	gliosis along resection cavity	SEEG	FCD type IIa
5	34.15	Male	FLE, right	negative	grid	FCD type IIb + DNH
6	47.63	Female	left hemisphere	ischemia	grid	Hypoxia
7	29.08	Male	F(T)LE, right	negative	grid	FCD type IIa
8	54.29	Male	FLE, left	negative	SEEG, grid	DNH
9	23.52	Male	FLE, left	FCD	SEEG, grid	DNH
10	16.65	Female	FLE, left/bilateral	T2 hyperintensity	SEEG	gliotic scar

DNH: diffuse neuronal heterotopia, FCD: focal cortical dysplasia, FLE: frontal lobe epilepsy, FTLE: frontotemporal lobe epilepsy, PLE: parietal lobe epilepsy.

**Table 2:** Overview of seizure frequency and medication.

Patient No.	Seizure Frequency	Seizure Type	Antiepileptic Drugs	
			Ineffective Medication	Current Medication
1	3/week	aura, FIAS, sleep related	BRV, CBZ, LTG, LEV, PER	LCM, ZNS
2	5–10/day	aura, FIAS	BRV, LCM	CBZ, LEV, TPM
3	3–4/month	G, TCS	CBZ, PB, OXC, VPA	LTG, LEV, TPM
4	2/week	aura, TCS	LEV, ZNS	BRV, CBZ, LCM
5	2–4/week	aura, TCS	LTG, LEV, VPA	LCM
6	2–7/week	TCS	CBZ, VPA	LEV, ZNS
7	3/week	aura, TCS	LCM, LTG, LEV, VPA	BRV, OXC, ZNS
8	2–3/week (2/year)	TCS (G)	LCM, TPM, VPA	LEV, PER
9	3/day	FIAS, TCS, sleep related	OXC, SUM	BRV, LCM, PER
10	2/week	aura, FAS	LCM, LTG, VPA	LEV, OXC

Seizure type—FAS: focal aware seizures, FIAS: focal impaired awareness seizures, G: generalized seizures, TCS: tonic clonic seizures; antiepileptic drugs—BRV: Brivaracetam, CBZ: Carbamazepin, LCM: Lacosamid, LTG: Lamotrigine, LEV: Levetiracetam, OXC: Oxcarbazepin, PB: Phenobarbital, PER: Perampanel, SUM: Sultiam, TPM: Topiramat, VPA: Valproat, ZNS: Zonisamid.

**Table 3:** Surgical and Epileptogenic Outcome.

Patient No.	Surgical Complications	New Postoperative Neurological Deficits	ILAE Outcome at One Year Follow-Up	ILAE Outcome at Latest Follow-Up
1	dislocation of boneflap, refixation required	-	1	1 (4 years)
2	-	-	1	1 (7 years)
3	-	-	2	2 (6 years)
4	-	slight coordination disturbance of the left side (<1 month)	1	1 (7 years)
5	-	-	1	1 (1 year)
6	-	-	4	4 (5 years)
7	CSF fistula, revision surgery required	-	1	1 (5 years)
8	-	-	3	3 (2 years)
9	-	transient sensoric aphasia (<3 weeks)	3	1 (2 years)
10	-	-	1	1 (4 years)

ILAE: International League Against Epilepsy.

**Table 4:** Visualized structures.

	Patient No.									
AR Visualization	1	2	3	4	5	6	7	8	9	10
Lesion	x	x	x	x	x	x	x	x	x	x
SEEG electrodes	x	x	x	x	-	-	-	-	x	x
Subdural grid electrodes	x	-	x	-	x	x	x	x	x	-
Seizure-related electrode contacts (onset, propagation)	x	-	-	-	x	x	-	x	x	x
Motor cortex	-	-	x	-	-	-	-	x	x	-
Cerebrum	x	-	-	-	x	-	x	x	x	x
CST	x	x	-	x	x	-	-	-	x	-
AF	x	x	-	x	-	-	-	-	x	-
IFOF	x	x	-	x	-	-	-	-	-	-
UF	x	x	-	-	-	-	-	-	-	-
fMRI language activation	-	x	-	-	-	-	-	-	-	x
Acquired subdural grid electrode contacts	x	-	-	-	x	-	x	x	-	-
SEEG trajectory	-	-	-	-	-	-	-	-	x	-

AF: arcuate fascicle, CST: corticospinal tracts, fMRI: functional magnetic resonance imaging, IFOF: inferior fronto-occipital fascicle, iUS: intraoperative ultrasound, UF: uncinate fascicle.

## Data Availability

The data in this study are available on request from the corresponding author. The data are not publicly available due to privacy restrictions.

## References

[1] Meola A., Cutolo F., Carbone M., Cagnazzo F., Ferrari M., Ferrari V. (2017). Augmented reality in neurosurgery: A systematic review. Neurosurg. Rev..

[2] Kelly P.J., Alker G.J., Goerss S. (1982). Computer-assisted stereotactic microsurgery for the treatment of intracranial neoplasms. Neurosurgery.

[3] Roberts D.W., Strohbehn J.W., Hatch J.F., Murray W., Kettenberger H. (1986). A frameless stereotaxic integration of computerized tomographic imaging and the operating microscope. J. Neurosurg..

[4] King A.P., Edwards P.J., Maurer C.R., de Cunha D.A., Hawkes D.J., Hill D.L., Gaston R.P., Fenlon M.R., Strong A.J., Chandler C.L. (1999). A system for microscope-assisted guided interventions. Stereotact. Funct. Neurosurg..

[5] Kiya N., Dureza C., Fukushima T., Maroon J.C. (1997). Computer navigational microscope for minimally invasive neurosurgery. Minim. Invasive Neurosurg..

[6] Cabrilo I., Bijlenga P., Schaller K. (2014). Augmented reality in the surgery of cerebral arteriovenous malformations: Technique assessment and considerations. Acta Neurochir..

[7] Cabrilo I., Bijlenga P., Schaller K. (2014). Augmented reality in the surgery of cerebral aneurysms: A technical report. Neurosurgery.

[8] Cabrilo I., Schaller K., Bijlenga P. (2015). Augmented reality-assisted bypass surgery: Embracing minimal invasiveness. World Neurosurg..

[9] Cannizzaro D., Zaed I., Safa A., Jelmoni A.J.M., Composto A., Bisoglio A., Schmeizer K., Becker A.C., Pizzi A., Cardia A. (2022). Augmented Reality in Neurosurgery, State of Art and Future Projections. A Systematic Review. Front. Surg..

[10] Mascitelli J.R., Schlachter L., Chartrain A.G., Oemke H., Gilligan J., Costa A.B., Shrivastava R.K., Bederson J.B. (2018). Navigation-Linked Heads-Up Display in Intracranial Surgery: Early Experience. Oper. Neurosurg..

[11] Sun G.C., Wang F., Chen X.L., Yu X.G., Ma X.D., Zhou D.B., Zhu R.Y., Xu B.N. (2016). Impact of Virtual and Augmented Reality Based on Intraoperative Magnetic Resonance Imaging and Functional Neuronavigation in Glioma Surgery Involving Eloquent Areas. World Neurosurg..

[12] Cabrilo I., Sarrafzadeh A., Bijlenga P., Landis B.N., Schaller K. (2014). Augmented reality-assisted skull base surgery. Neurochirurgie.

[13] Carl B., Bopp M., Benescu A., Sass B., Nimsky C. (2020). Indocyanine Green Angiography Visualized by Augmented Reality in Aneurysm Surgery. World Neurosurg..

[14] Carl B., Bopp M., Sass B., Pojskic M., Voellger B., Nimsky C. (2020). Spine Surgery Supported by Augmented Reality. Glob. Spine J..

[15] Carl B., Bopp M., Sass B., Voellger B., Nimsky C. (2019). Implementation of augmented reality support in spine surgery. Eur. Spine J..

[16] Devinsky O., Hesdorffer D.C., Thurman D.J., Lhatoo S., Richerson G. (2016). Sudden unexpected death in epilepsy: Epidemiology, mechanisms, and prevention. Lancet Neurol..

[17] Kwan P., Brodie M.J. (2000). Early identification of refractory epilepsy. N. Engl. J. Med..

[18] Thurman D.J., Beghi E., Begley C.E., Berg A.T., Buchhalter J.R., Ding D., Hesdorffer D.C., Hauser W.A., Kazis L., Kobau R. (2011). Standards for epidemiologic studies and surveillance of epilepsy. Epilepsia.

[19] Wiebe S., Blume W.T., Girvin J.P., Eliasziw M. (2001). Effectiveness and Efficiency of Surgery for Temporal Lobe Epilepsy Study. Group A randomized, controlled trial of surgery for temporal-lobe epilepsy. N. Engl. J. Med..

[20] McIntosh A.M., Averill C.A., Kalnins R.M., Mitchell L.A., Fabinyi G.C., Jackson G.D., Berkovic S.F. (2012). Long-term seizure outcome and risk factors for recurrence after extratemporal epilepsy surgery. Epilepsia.

[21] Delev D., Oehl B., Steinhoff B.J., Nakagawa J., Scheiwe C., Schulze-Bonhage A., Zentner J. (2019). Surgical Treatment of Extratemporal Epilepsy: Results and Prognostic Factors. Neurosurgery.

[22] Cascino G.D. (2004). Surgical Treatment for Extratemporal Epilepsy. Curr. Treat. Options Neurol..

[23] Roper S.N. (2009). Surgical treatment of the extratemporal epilepsies. Epilepsia.

[24] Wurm G., Ringler H., Knogler F., Schnizer M. (2003). Evaluation of neuronavigation in lesional and non-lesional epilepsy surgery. Comput. Aided Surg..

[25] Wellmer J., von der Groeben F., Klarmann U., Weber C., Elger C.E., Urbach H., Clusmann H., von Lehe M. (2012). Risks and benefits of invasive epilepsy surgery workup with implanted subdural and depth electrodes. Epilepsia.

[26] Rosenow F., Luders H. (2001). Presurgical evaluation of epilepsy. Brain.

[27] Kral T., Clusmann H., Urbach J., Schramm J., Elger C.E., Kurthen M., Grunwald T. (2002). Preoperative evaluation for epilepsy surgery (Bonn Algorithm). Zentralbl. Neurochir..

[28] Shah A.K., Mittal S. (2014). Invasive electroencephalography monitoring: Indications and presurgical planning. Ann. Indian Acad. Neurol..

[29] Chamoun R.B., Nayar V.V., Yoshor D. (2008). Neuronavigation applied to epilepsy monitoring with subdural electrodes. Neurosurg. Focus..

[30] Kamida T., Anan M., Shimotaka K., Abe T., Fujiki M., Kobayashi H. (2010). Visualization of subdural electrodes with fusion CT scan/MRI during neuronavigation-guided epilepsy surgery. J. Clin. Neurosci..

[31] Maslarova A., Zhao Y., Rosch J., Dorfler A., Coras R., Blumcke I., Lang J., Schmidt M., Hamer H.M., Reindl C. (2023). Surgical planning, histopathology findings and postoperative outcome in MR-negative extra-temporal epilepsy using intracranial EEG, functional imaging, magnetoencephalography, neuronavigation and intraoperative MRI. Clin. Neurol. Neurosurg..

[32] Nimsky C., Buchfelder M. (2003). Neuronavigation in epilepsy surgery. Arq. Neuropsiquiatr..

[33] Roethe A.L., Rosler J., Misch M., Vajkoczy P., Picht T. (2022). Augmented reality visualization in brain lesions: A prospective randomized controlled evaluation of its potential and current limitations in navigated microneurosurgery. Acta Neurochir..

[34] Leger E., Drouin S., Collins D.L., Popa T., Kersten-Oertel M. (2017). Quantifying attention shifts in augmented reality image-guided neurosurgery. Healthc. Technol. Lett..

[35] Carl B., Bopp M., Gjorgjevski M., Nimsky C. (2018). Navigation-Supported Stereotaxy by Applying Intraoperative Computed Tomography. World Neurosurg..

[36] Carl B., Bopp M., Sass B., Nimsky C. (2018). Intraoperative computed tomography as reliable navigation registration device in 200 cranial procedures. Acta Neurochir..

[37] Wieser H.G., Blume W.T., Fish D., Goldensohn E., Hufnagel A., King D., Sperling M.R., Luders H., Pedley T.A., Commission on Neurosurgery of the International League AgainstEpilepsy (2001). ILAE Commission Report. Proposal for a new classification of outcome with respect to epileptic seizures following epilepsy surgery. Epilepsia.

[38] Gajdos M., Riha P., Kojan M., Dolezalova I., Mutsaerts H., Petr J., Rektor I. (2021). Epileptogenic zone detection in MRI negative epilepsy using adaptive thresholding of arterial spin labeling data. Sci. Rep..

[39] Tellez-Zenteno J.F., Hernandez Ronquillo L., Moien-Afshari F., Wiebe S. (2010). Surgical outcomes in lesional and non-lesional epilepsy: A systematic review and meta-analysis. Epilepsy Res..

[40] Hader W.J., Mackay M., Otsubo H., Chitoku S., Weiss S., Becker L., Snead O.C., Rutka J.T. (2004). Cortical dysplastic lesions in children with intractable epilepsy: Role of complete resection. J. Neurosurg..

[41] Neuloh G., Bien C.G., Clusmann H., von Lehe M., Schramm J. (2010). Continuous motor monitoring enhances functional preservation and seizure-free outcome in surgery for intractable focal epilepsy. Acta Neurochir..

[42] Delev D., Send K., Malter M., Ormond D.R., Parpaley Y., von Lehe M., Schramm J., Grote A. (2015). Role of Subdural Interhemispheric Electrodes in Presurgical Evaluation of Epilepsy Patients. World Neurosurg..

[43] So E.L., Lee R.W. (2014). Epilepsy surgery in MRI-negative epilepsies. Curr. Opin. Neurol..

[44] Hedegard E., Bjellvi J., Edelvik A., Rydenhag B., Flink R., Malmgren K. (2014). Complications to invasive epilepsy surgery workup with subdural and depth electrodes: A prospective population-based observational study. J. Neurol. Neurosurg. Psychiatry.

[45] McGonigal A., Bartolomei F., Regis J., Guye M., Gavaret M., Trebuchon-Da Fonseca A., Dufour H., Figarella-Branger D., Girard N., Peragut J.C. (2007). Stereoelectroencephalography in presurgical assessment of MRI-negative epilepsy. Brain.

[46] Carl B., Bopp M., Sass B., Pojskic M., Gjorgjevski M., Voellger B., Nimsky C. (2019). Reliable navigation registration in cranial and spine surgery based on intraoperative computed tomography. Neurosurg. Focus.

[47] Watanabe Y., Fujii M., Hayashi Y., Kimura M., Murai Y., Hata M., Sugiura A., Tsuzaka M., Wakabayashi T. (2009). Evaluation of errors influencing accuracy in image-guided neurosurgery. Radiol. Phys. Technol..

[48] Stieglitz L.H., Fichtner J., Andres R., Schucht P., Krahenbuhl A.K., Raabe A., Beck J. (2013). The silent loss of neuronavigation accuracy: A systematic retrospective analysis of factors influencing the mismatch of frameless stereotactic systems in cranial neurosurgery. Neurosurgery.

[49] Kantelhardt S.R., Gutenberg A., Neulen A., Keric N., Renovanz M., Giese A. (2015). Video-Assisted Navigation for Adjustment of Image-Guidance Accuracy to Slight Brain Shift. Oper. Neurosurg..

[50] Steinmeier R., Rachinger J., Kaus M., Ganslandt O., Huk W., Fahlbusch R. (2000). Factors influencing the application accuracy of neuronavigation systems. Stereotact. Funct. Neurosurg..

[51] Nimsky C., Ganslandt O., Cerny S., Hastreiter P., Greiner G., Fahlbusch R. (2000). Quantification of, visualization of, and compensation for brain shift using intraoperative magnetic resonance imaging. Neurosurgery.

[52] Poggi S., Pallotta S., Russo S., Gallina P., Torresin A., Bucciolini M. (2003). Neuronavigation accuracy dependence on CT and MR imaging parameters: A phantom-based study. Phys. Med. Biol..

[53] Hastreiter P., Rezk-Salama C., Soza G., Bauer M., Greiner G., Fahlbusch R., Ganslandt O., Nimsky C. (2004). Strategies for brain shift evaluation. Med. Image Anal..

[54] Nimsky C., Ganslandt O., Hastreiter P., Fahlbusch R. (2001). Intraoperative compensation for brain shift. Surg. Neurol..

[55] Oertel J., Gaab M.R., Runge U., Schroeder H.W., Wagner W., Piek J. (2004). Neuronavigation and complication rate in epilepsy surgery. Neurosurg. Rev..

[56] Centeno R.S., Yacubian E.M., Sakamoto A.C., Ferraz A.F., Junior H.C., Cavalheiro S. (2006). Pre-surgical evaluation and surgical treatment in children with extratemporal epilepsy. Childs Nerv. Syst..

[57] Sommer B., Grummich P., Coras R., Kasper B.S., Blumcke I., Hamer H.M., Stefan H., Buchfelder M., Roessler K. (2013). Integration of functional neuronavigation and intraoperative MRI in surgery for drug-resistant extratemporal epilepsy close to eloquent brain areas. Neurosurg. Focus.

[58] Sommer B., Roessler K., Rampp S., Hamer H.M., Blumcke I., Stefan H., Buchfelder M. (2016). Magnetoencephalography-guided surgery in frontal lobe epilepsy using neuronavigation and intraoperative MR imaging. Epilepsy Res..

[59] Bien C.G., Szinay M., Wagner J., Clusmann H., Becker A.J., Urbach H. (2009). Characteristics and surgical outcomes of patients with refractory magnetic resonance imaging-negative epilepsies. Arch. Neurol..

[60] Cho D.Y., Lee W.Y., Lee H.C., Chen C.C., Tso M. (2005). Application of neuronavigator coupled with an operative microscope and electrocorticography in epilepsy surgery. Surg. Neurol..

[61] Brinker T., Arango G., Kaminsky J., Samii A., Thorns U., Vorkapic P., Samii M. (1998). An experimental approach to image guided skull base surgery employing a microscope-based neuronavigation system. Acta Neurochir..

[62] Kajiwara K., Nishizaki T., Ohmoto Y., Nomura S., Suzuki M. (2003). Image-guided transsphenoidal surgery for pituitary lesions using Mehrkoordinaten Manipulator (MKM) navigation system. Minim. Invasive Neurosurg..

[63] Meola A., Chang S.D. (2018). Letter: Navigation-Linked Heads-Up Display in Intracranial Surgery: Early Experience. Oper. Neurosurg..

[64] Edwards P.J., Johnson L.G., Hawkes D.J., Fenlon M.R., Strong A.J., Gleeson M.J. (2004). Clinical Experience and Perception in Stereo Augmented Reality Surgical Navigation.

[65] Su Y., Sun Y., Hosny M., Gao W., Fu Y. (2022). Facial landmark-guided surface matching for image-to-patient registration with an RGB-D camera. Int. J. Med. Robot..

[66] West J.B., Fitzpatrick J.M., Toms S.A., Maurer C.R., Maciunas R.J. (2001). Fiducial point placement and the accuracy of point-based, rigid body registration. Neurosurgery.

[67] Mitsui T., Fujii M., Tsuzaka M., Hayashi Y., Asahina Y., Wakabayashi T. (2011). Skin shift and its effect on navigation accuracy in image-guided neurosurgery. Radiol. Phys. Technol..

[68] Pfisterer W.K., Papadopoulos S., Drumm D.A., Smith K., Preul M.C. (2008). Fiducial versus nonfiducial neuronavigation registration assessment and considerations of accuracy. Neurosurgery.

[69] Rachinger J., von Keller B., Ganslandt O., Fahlbusch R., Nimsky C. (2006). Application accuracy of automatic registration in frameless stereotaxy. Stereotact. Funct. Neurosurg..

[70] Letteboer M.M., Willems P.W., Viergever M.A., Niessen W.J. (2005). Brain shift estimation in image-guided neurosurgery using 3-D ultrasound. IEEE Trans. Biomed. Eng..

[71] Reinertsen I., Lindseth F., Askeland C., Iversen D.H., Unsgard G. (2014). Intra-operative correction of brain-shift. Acta Neurochir..

[72] Saß B., Carl B., Pojskic M., Nimsky C., Bopp M. (2020). Navigated 3D Ultrasound in Brain Metastasis Surgery: Analyzing the Differences in Object Appearances in Ultrasound and Magnetic Resonance Imaging. Appl. Sci..

[73] Bopp M.H.A., Grote A., Gjorgjevski M., Pojskic M., Sass B., Nimsky C. (2024). Enabling Navigation and Augmented Reality in the Sitting Position in Posterior Fossa Surgery Using Intraoperative Ultrasound. Cancers.

[74] Nakajima S., Atsumi H., Kikinis R., Moriarty T.M., Metcalf D.C., Jolesz F.A., Black P.M. (1997). Use of cortical surface vessel registration for image-guided neurosurgery. Neurosurgery.

[75] Bopp M.H.A., Corr F., Sass B., Pojskic M., Kemmling A., Nimsky C. (2022). Augmented Reality to Compensate for Navigation Inaccuracies. Sensors.

[76] Thavarajasingam S.G., Vardanyan R., Arjomandi Rad A., Thavarajasingam A., Khachikyan A., Mendoza N., Nair R., Vajkoczy P. (2022). The use of augmented reality in transsphenoidal surgery: A systematic review. Br. J. Neurosurg..

[77] Bopp M.H.A., Sass B., Pojskic M., Corr F., Grimm D., Kemmling A., Nimsky C. (2022). Use of Neuronavigation and Augmented Reality in Transsphenoidal Pituitary Adenoma Surgery. J. Clin. Med..

[78] Pojskic M., Bopp M., Sass B., Nimsky C. (2024). Single-Center Experience of Resection of 120 Cases of Intradural Spinal Tumors. World Neurosurg..

[79] Pojskic M., Bopp M.H.A., Sass B., Nimsky C. (2024). Single-Center Experience in Microsurgical Resection of Acoustic Neurinomas and the Benefit of Microscope-Based Augmented Reality. Medicina.

[80] Ahmadipour Y., Lemonas E., Maslehaty H., Goericke S., Stuck B.A., El Hindy N., Sure U., Mueller O. (2016). Critical analysis of anatomical landmarks within the sphenoid sinus for transsphenoidal surgery. Eur. Arch. Otorhinolaryngol..

